# Impact of Microbiome‐Immune Interplay on Cognitive Impairment: An Investigation from the MiaGB Cohort

**DOI:** 10.1002/alz70855_102998

**Published:** 2025-12-23

**Authors:** Santosh Kumar Prajapati, Rohit Shukla, Vivek Kumar, Dhananjay Yadav, Lalitha Lekkala, Charles Szekeres, Yonggang Ma, Shalini Jain, Hariom Yadav

**Affiliations:** ^1^ Center for Microbiome Research, Microbiomes Institute, University of South Florida, Tampa, FL, USA; ^2^ College of Medicine, Molecular Medicine, University of South Florida, Tampa, FL, USA; ^3^ College of Medicine, Molecular Pharmacology & Physiology, University of South Florida, Tampa, FL, USA; ^4^ Department of Neurosurgery and Brain Repair, Morsani College of Medicine, University of South Florida, Tampa, FL, USA

## Abstract

**Background:**

Alzheimer's disease (AD) pathogenesis has been linked to the microbiota‐immune‐brain axis; however, the relationship between gut microbiota, immune activity, and cognitive impairment remains unclear. Thus, this study examines the connection between intestinal microbial composition, immune cell phenotype, and cognitive function in older adults.

**Method:**

Data and biological samples were obtained from participants aged ≥60 years (Control, n = 30; mild cognitive impairment (MCI), n = 30) from the MiaGB (Microbiome in Aging Gut and Brain) consortium, a multi‐site, clinical study. Cognitive function was assessed using Montreal Cognitive Assessment (MoCA) scores, immunophenotyping through flow cytometry, stool microbiome analysis using whole‐genome metagenomics, and bulk transcriptomics analysis was carried out.

**Results:**

The abundance of immune cells such as granulocytes, lymphocytes, T‐cells, and NK cells was significantly decreased in MCI group. Interestingly, the levels of CD4+ were reduced while CD8+ cells increased in MCI participants compared to controls. Microbial profiling revealed distinct bacterial signatures, with MCI participants showing higher relative abundances of *Eubacterium hallii, Parabacteroides distasonis, Eggerthella_sp_CAG_298, Dorea formicigenerans* and *Alistipes finegldii*. Differential expression analysis of transcriptomics data identified 1632 upregulated and 240 downregulated genes. Gene ontology and pathway analysis revealed that upregulated genes are involved in several immune functions such as response to stimulus, adaptive immune response, lymphocyte, and T cell activation, while downregulated genes are linked to nervous system functions and signaling processes such as neuron projection. Transcriptomics analysis further highlighted that several downregulated genes are involved in the key pathways that participate in the neural functions.

**Conclusion:**

These distinct bacteria, immune cells, and gene expression profiles suggest that alterations in immune cell populations, gene expression, and gut microbiota are associated with cognitive function in aging, highlighting potential interactions between the microbiota‐immune‐brain axis and cognitive impairment.

